# Draft Genome Sequence of *Aspergillus niger* Strain An76

**DOI:** 10.1128/genomeA.01700-15

**Published:** 2016-02-18

**Authors:** Weili Gong, Zhi Cheng, Huaiqiang Zhang, Lin Liu, Peiji Gao, Lushan Wang

**Affiliations:** The State Key Laboratory of Microbial Technology, Shandong University, Jinan, Shandong, China

## Abstract

The filamentous fungus *Aspergillus niger* has become one of the most important fungi in industrial biotechnology, and it can efficiently secrete both polysaccharide-degrading enzymes and organic acids. We report here the 6,074,961,332-bp draft sequence of *A. niger* strain An76, and the findings provide important information related to its lignocellulose-degrading ability.

## GENOME ANNOUNCEMENT

*Aspergillus niger* is distributed widely and abundantly in nature and displays diverse phenotypes (1, 2). Recently, it has become one of the most frequently used fungi in industrial biotechnology, and it can produce polysaccharide-degrading enzymes (especially xylanases, pectinases, and amylases) and organic acids (mainly citric acid) (3–5). *A. niger* strain An76 was derived from the rapidly growing and high-xylanase-producing strain *A. niger* C-2. An76 maintains the essential lignocellulose-degrading feature of the original strain and obtains the elevated capacity to synthesize β-xylanase and β-xylosidase (6). In the previous study, the dynamic changes in xylanases and β-1,4-endoglucanases secreted by *A. niger* An76 in response to hydrolysates of lignocellulose polysaccharide have been shown by dynamic zymography (7). However, it was difficult to research the lignocellulose-degrading mechanism of this strain due to the lack of its genome sequence data. Therefore, the genome sequence of *A. niger* An76 was determined in order to facilitate an understanding of its lignocellulose degradation strategy.

The Illumina HiSeq platform at Shanghai Majorbio was utilized to sequence the genome of An76. One 300-bp paired-end (PE) library was prepared for sequencing, generating 5,932.5 Mb of raw data (read length, 6,074,961,332 bp). The reads were adapter clipped, non-AGCT bases on the 5′ ends were removed before being cut and quality trimmed (sequencing quality values, <Q20), N-containing (>10%) reads were removed, and small fragments (length, <25 bp) were abandoned following adapter clipping and quality trimming. Next, the high-quality reads (5,377.6 Mb) was assessed using k-mer-counting tools, indicating a genome size of approximately 30 Mb, with low heterozygosity (<0.1%) and normal G+C-depth distribution (44% to ~56%). The 30 Mb of error-corrected reads were assembled with SOAP*denovo* version 2.04, and the partial gap and incorrect bases in the assembled result were supplemented and corrected by GapCloser version 1.12. The gene prediction was performed by Augustus version 2.5.5, and the predicted genes were annotated using the NCBI nr, GO, STRING, and KEGG databases.

The draft genome contains 669 scaffolds covering 34,880,193 bp and 487 large contigs (>1,000 bp), with a total length of 34,658,779 bp. The *N*_50_ contig length is 300,933 bp, and the *N*_90_ contig length is 73,290 bp. The G+C content is 47%.

The *A. niger* draft genome includes 10,373 protein-coding genes, of which 79 genes are annotated to encode glycoside hydrolase (GH). The analysis using the KEGG pathway revealed that most of these protein-encoding genes were involved in glycolysis/gluconeogenesis, the tricarboxylic acid (TCA) cycle, the pentose phosphate pathway, and fatty acid biosynthesis. There is also a decent part of these genes taking part in galactose, mannose, fructose, sucrose, and starch metabolism.

The genome sequence of *A. niger* An76 is considerably valuable for further functional genome study and can shed light on the investigation of transcriptional regulation on lignocellulose degradation. Comprehensive analysis of this specific strain’s genome will be reported in the future.

### Nucleotide sequence accession number.

The shotgun genome project has been deposited in the DNA Data Bank of Japan (DDBJ) under the accession no. BCMY00000000. The version described in this paper is the first version.
